# The use of three-dimensional imaging for R0 resection of a left upper lobe adenocarcinoma after coronary artery bypass surgery

**DOI:** 10.1016/j.xjtc.2022.04.028

**Published:** 2022-05-06

**Authors:** Shelby Shamir, Richard S. Lazzaro, Matthew L. Inra

**Affiliations:** a Department of Cardiothoracic Surgery, Lenox Hill Hospital – Northwell Health, New York, NY; b Division of Thoracic Surgery, Department of Cardiothoracic Surgery, Lenox Hill Hospital – Northwell Health, New York, NY; c Division of Thoracic Surgery, Department of Surgery, Robert Wood Johnson Foundation, Long Branch, NJ

**Figure:**
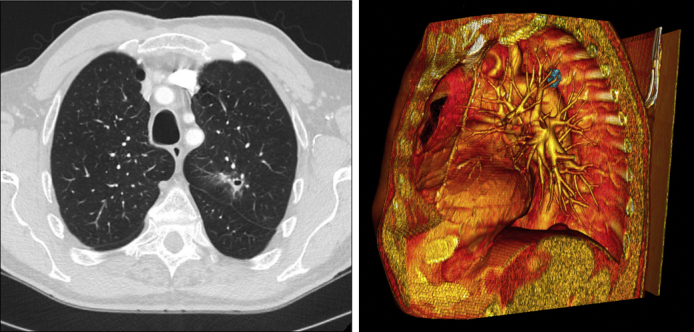
Two-dimensional computed tomography of nodule and 3D reconstruction of patent LITA graft and nodule (blue).

**Central Message**

We describe preoperative considerations and imaging for successfully preparing for and performing video-assisted thoracoscopic surgery, robotic-assisted left upper lobectomy in the presence of a patent LITA to LAD bypass graft.

An 85-year-old man with coronary artery disease (CAD) and history of left internal thoracic artery (LITA) to left anterior descending (LAD) bypass via left thoracotomy presented for evaluation of a left upper lobe pulmonary nodule. Differential diagnosis and therapeutic options were discussed, including stereotactic radiotherapy. After preoperative staging and medical evaluation, he was deemed an operative candidate. His forced expiratory volume at 1 second and diffusing capacity for carbon monoxide were 107% and 88% of predicted, respectively, and cardiac evaluation was unremarkable. The patient elected to undergo surgery. He consented to left video-assisted thoracoscopic surgery, robotic-assisted upper lobe wedge resection with completion lobectomy, and mediastinal lymph node dissection for malignancy confirmed intraoperatively. Risks of the operation and to the graft were discussed. When consenting to the operation, the patient verbally consented to the use of deidentified pictures and videos for academic purposes.

Preoperatively, 3-dimensional (3D) reconstruction of his 2-dimensional (2D) computed tomography with contrast was performed using a DICOM viewer (Figure 1) and were converted into a movie file (Video 1). This reconstruction helped identify the patent bypass graft and its relationship to other known structures. The patient underwent this procedure in a reoperative field. After adhesiolysis, the graft was identified by separating the mediastinal surface of the upper lobe from the heart and graft using a no-touch technique. The distal extent of the graft was identified on imaging (Figure 1), aiding the initial dissection. After freeing the surface of the lung from the graft, wedge resection and completion lobectomy and mediastinal lymph node dissection were performed for definitive cancer treatment and staging (Figure 2). There were no intraoperative complications.

**Figure 1:**
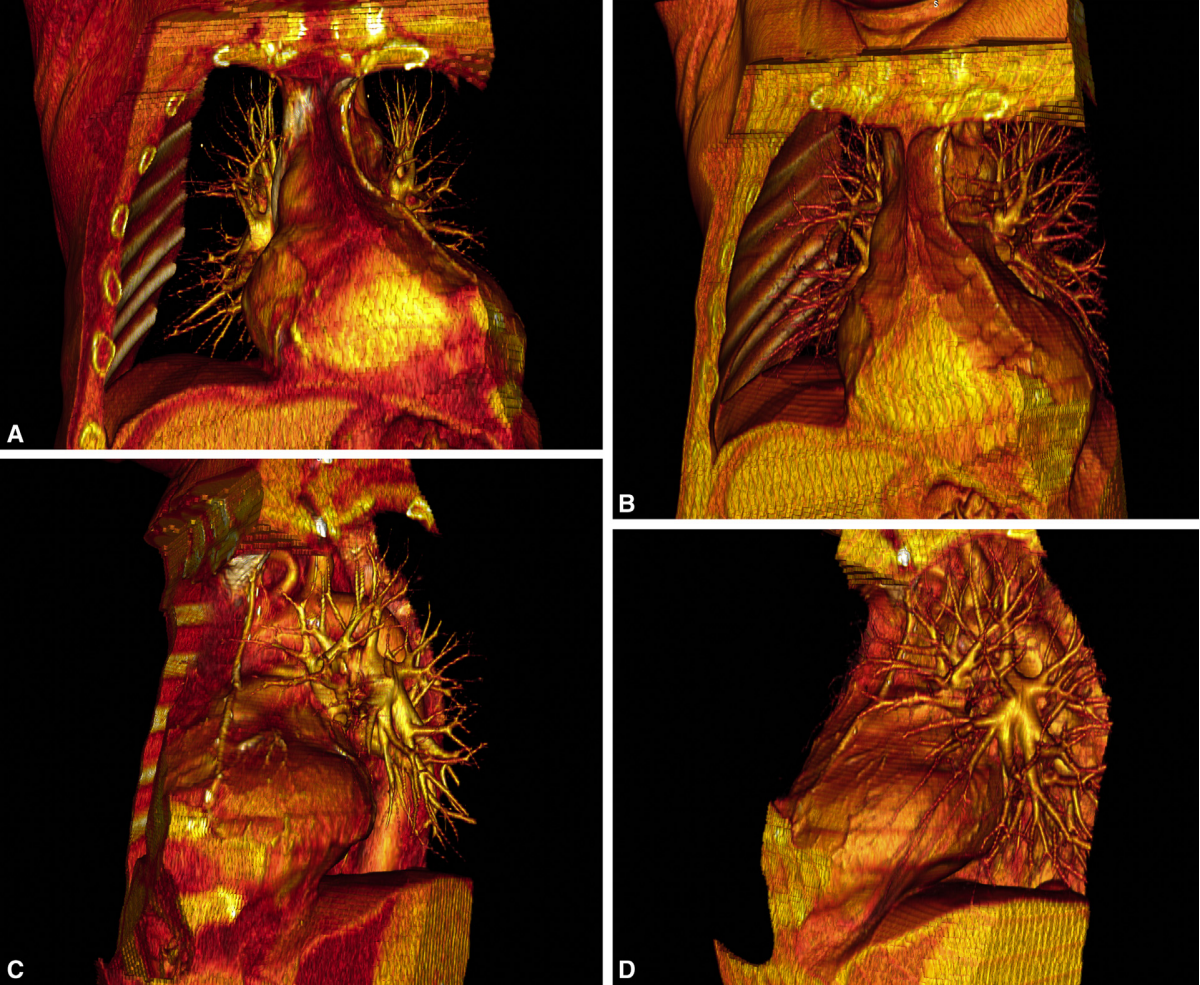
Three-dimensional still pictures of the patient's reconstruction from the 2D computed tomography angiography. A-D, The graft's path through the mediastinum on the left anterior surface of the heart and its relationship to the left upper lobe hilar structures from the most anterior view (A) to a left posterolateral view (D).

**Figure 2:**
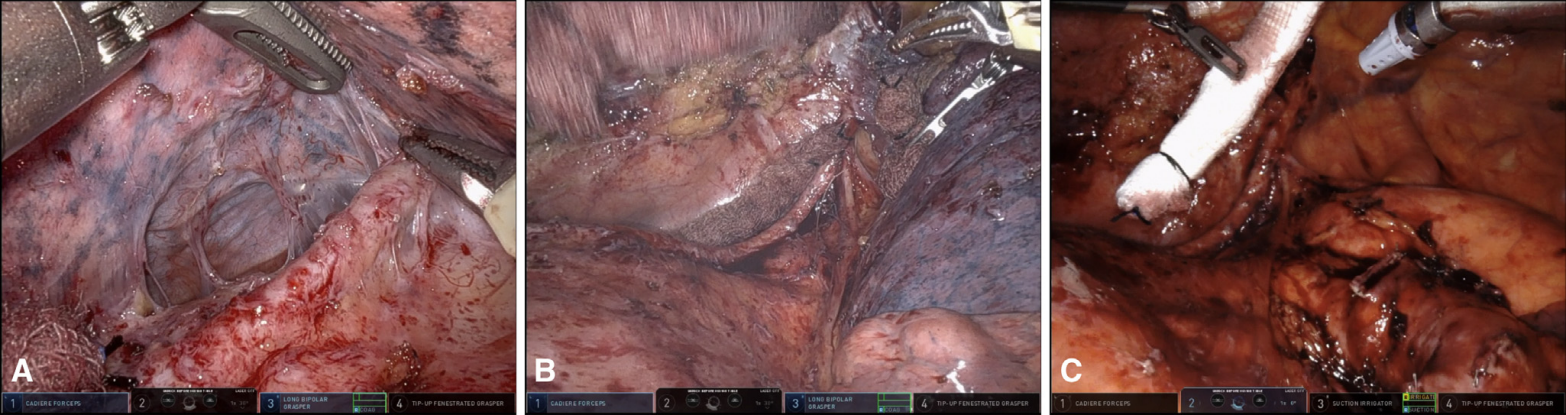
A-C, Intraoperative pictures identifying the LITA graft at its distal extent (A), at the completion of dissection of the left upper lobe from the mediastinum with the graft intact (B), and at the completion of the left upper lobectomy with the uninjured graft (C). *LITA*, Left internal thoracic artery.

The patient's chest tube was removed on postoperative day 1, and dual antiplatelet therapy was restarted. He was discharged home on postoperative day 2 without complication. His pathological stage was IA adenocarcinoma. He is doing well 6 months postoperatively with no sign of recurrence and a patent graft on his first surveillance scan.

Smoking is a risk factor for both lung cancer and CAD. Therefore, it is not surprising that patients with resectable lung cancers may have patent LITA to LAD bypass grafts. Previous LITA harvest and LAD anastomosis increase the complexity of the operation due to adhesion formation, putting the graft at risk.^1^, ^2^, ^3^ The presence of a LITA to LAD bypass graft, however, should not preclude surgical treatment for resectable lung cancer in an operable patient.^1^^,^^2^ This case highlights the importance of preoperative planning for completion of a complex lung resection using advanced imaging techniques and a robotic-assisted surgical approach.

Although we do not use 3D reconstruction for every lung resection, this case is an example of an operation where more preoperative preparation is beneficial. We developed an imaging protocol at our institution allowing us to create 3D reconstructions from 2D images, specifically for delineation of vascular anatomy. A 256-slice computed tomography from the lung apices to the costophrenic angles is performed at deep inspiration. The region of interest is placed over the pulmonary trunk with a 60 Hounsfield unit threshold used to initiate the scan after a 6-second delay. Contrast is injected at 5 mL/sec for 100 mL with a retrospectively obtained electrocardiogram gated helical acquisition. This technique provides good separation of the pulmonary and systemic circulation. Using a DICOM viewer, we create 3D reconstructions to examine anatomy from all angles. Tissues of different densities can be added and subtracted as well to display their spatial relationships. The importance of 3D imaging in surgery has been described,^4^ and we believe it provides a better understanding of anatomy preoperatively showing all structures in a completely 3D spatial relationship.

This procedure has 2 principles: preserve the graft and achieve an R0 resection with appropriate lymph node staging for lung cancer. We were able to achieve the 2 principles of the operation while staying completely thoracoscopic because of our preoperative imaging and planning. We started dissection at the distal aspect of the graft, proceeding proximally. Our reconstruction clearly defined the distal extent of the graft, allowing us to anticipate its location intraoperatively. By starting with the least dense adhesions in a known area, and progressing to unknown, we successfully achieved the 2 principles. Our reconstruction made the unknown known by revealing the spatial relationship of the graft to other structures preoperatively, leading to anticipation of the graft's location and confident, safe dissection. Other adjuncts for safety can be considered for this procedure, such as groin prepping and cardiopulmonary bypass on standby.

Preoperative imaging and planning are crucial for complex operations. Three-dimensional reconstructions are excellent complements to 2D imaging and should be considered for complex lung resections for surgeons at all stages of their career.

## Supplementary Data

Video 1 Three-dimensional reconstruction showing the LITA on the left anterior surface of the heart. The stored movie file from the 3D reconstruction allows examination of the whole graft and its relationship to the other structures in the left hemithorax. The image is rotated in real time to follow the course of the graft from its most proximal extent to most distal. The tissue is cropped out and back in to give appropriate views, and the lung parenchyma can be introduced as well to show relative anatomy. Video available at: https://www.jtcvs.org/article/S2666-2507(22)00267-X/fulltext.

## References

[1] Wei B., Broussard B., Bryant A., Linsky P., Minnich D., Cerfolio R.J. (2015). Left upper lobectomy after coronary artery bypass grafting. J Thorac Cardiovasc Surg.

[2] Shah A.A., Worni M., Onaitis M.W., Balderson S.S., Harpole D.H., D’Amico T.A. (2014). Thorascopic left upper lobectomy in patients with internal mammary artery coronary bypass grafts. Ann Thorac Surg.

[3] Papiashvili M., Deviri E., Bar I., Sasson L. (2015). Lobectomy for non-small cell lung cancer after coronary artery bypass grafting surgery. Isr Med Assoc J.

[4] Simpfendörfer T., Ziyao L., Gasch C., Drosdzol F., Fangerau M., Müller M. (2017). Three-dimensional reconstruction of preoperative imaging improves surgical success in laparoscopy. J Laparoendosc Adv Surg Tech A.

