# Supplementary figures and images for: The use of three-dimensional imaging for R0 resection of a left upper lobe adenocarcinoma after coronary artery bypass surgery
Source: JTCVS Tech. 2022 May 6;14:141–3. doi: 10.1016/j.xjtc.2022.04.028 (PMC9366622; doi:10.1016/j.xjtc.2022.04.028)

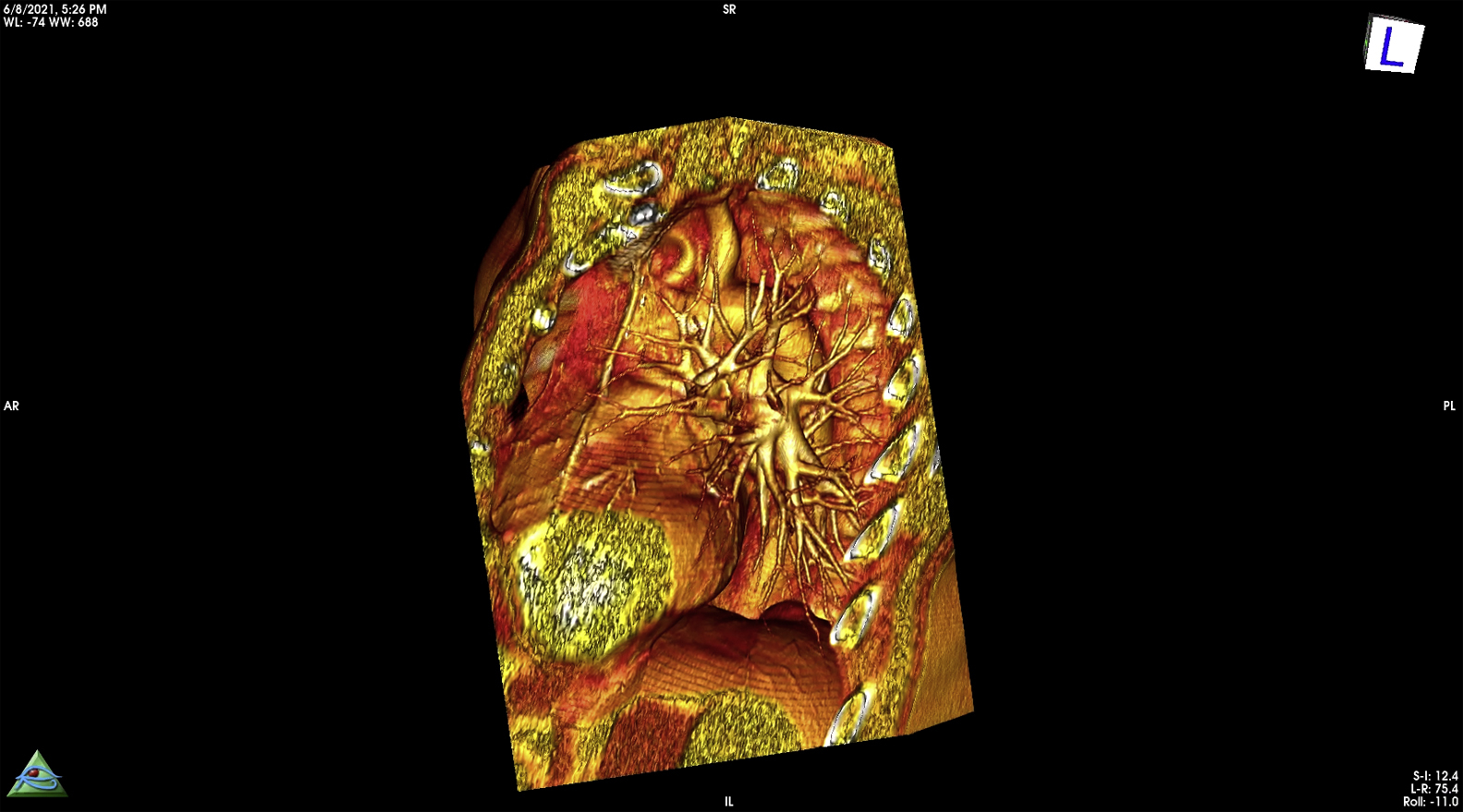

Supplement: Video 1 — Three-dimensional reconstruction showing the LITA on the left anterior surface of the heart. The stored movie file from the 3D reconstruction allows examination of the whole graft and its relationship to the other structures in the left hemithorax. The image is rotated in real time to follow the course of the graft from its most proximal extent to most distal. The tissue is cropped out and back in to give appropriate views, and the lung parenchyma can be introduced as well to show relative anatomy. Video available at: https://www.jtcvs.org/article/S2666-2507(22)00267-X/fulltext. [file fx2.jpg]
